# Determinants of Health-Promoting Behaviors Among Indonesian Adolescents Living in Child Welfare Institutions: Cross-Sectional Survey Study

**DOI:** 10.2196/75024

**Published:** 2025-09-17

**Authors:** Aloysia Ispriantari, Hyejung Lee, Sue Kim, Hyeonkyeong Lee, Anna Lee, Chang Park

**Affiliations:** 1 College of Nursing Yonsei University Seoul Republic of Korea; 2 Department of Nursing Faculty of Health Sciences Institute of Technology and Health Science RS dr Soepraoen Malang Indonesia; 3 Mo-Im Kim Nursing Research Institute College of Nursing Yonsei University Seoul Republic of Korea; 4 College of Nursing University of Illinois Chicago Chicago United States

**Keywords:** adolescents, health-promoting behaviors, child welfare institutions, Indonesia, health promotion model, structural equation model

## Abstract

**Background:**

Adolescents in child welfare institutions often face inadequate facility resources, limited caregiver support, and restricted access to health care services. These obstacles impede their physical, mental, and social development during adolescence, resulting in significant health vulnerabilities. Engaging in health-promoting behaviors (HPBs) can enhance their overall health and quality of life, potentially contributing to improved long-term well-being.

**Objective:**

This study aimed to identify the HPBs of adolescents in child welfare institutions in Indonesia and explore the determinants influencing these behaviors using the health promotion model.

**Methods:**

This study used a cross-sectional survey design. Adolescents living in welfare institutions were recruited from January 14, 2024, to February 3, 2024. After obtaining institutional review board approval, 6 research assistants visited 17 institutions in Malang and collected data from participants who provided their consent using tablet PCs that linked to the questionnaire. The variables studied included HPBs, health literacy, self-esteem, perceived barriers to action, perceived self-efficacy, and social support. Multivariate structural analysis was conducted using SPSS Statistics (version 26.0; IBM Corp) and SPSS Amos (version 26.0; IBM Corp).

**Results:**

A total of 276 adolescents participated in this study. Adolescents’ HPBs differed significantly based on age group (*P*=.03), educational level (*P*=.04), duration of stay in child welfare institutions (*P*=.03), and the institutions’ accreditation level (*P*=.02). In the final model, perceived self-efficacy (β=0.538; *P*<.001) and social support (β=0.256; *P*<.001) together accounted for 47.9% of the variance in HPBs. Self-esteem was positively correlated with perceived self-efficacy (β=0.184; *P*<.001) and social support (β=0.303; *P*<.001) but negatively correlated with perceived barriers to action (β=−0.194; *P*<.01). Health literacy was also negatively correlated with perceived barriers to action (β=−0.234; *P*<.001). Self-esteem indirectly affected HPBs through perceived self-efficacy (β=0.099; *P*<.01) and social support (β=0.078; *P*<.001).

**Conclusions:**

To improve the HPBs of adolescents living in child welfare institutions, their self-esteem needs to be increased to further enhance their self-efficacy and social support. Careful attention and monitoring of HPBs among these adolescents may lead to better health outcomes and support their transition from child welfare institutions to the broader community.

## Introduction

### Background

Adolescents living in child welfare institutions—commonly referred to as orphanages or childcare institutions—face unique challenges that may adversely affect their overall health and well-being. These adolescents are more prone to experiencing physical, mental, and social difficulties than their peers living in family environments [1]. Furthermore, these challenges can lead to hopelessness and hinder their ability to thrive [2].

Health-promoting behaviors (HPBs) refer to individuals’ actions and choices to improve their health and prevent the occurrence of diseases [3]. The World Health Organization emphasizes HPBs as a key strategy for improving health outcomes and enhancing quality of life [4]. During adolescence, engagement in HPBs is linked to improved academic performance, stronger interpersonal relationships, and a lower risk of future chronic diseases [5]. Thus, adopting HPBs during adolescence not only is beneficial for the present but also serves as a crucial investment in their future health and well-being.

Although HPBs have many advantages, barriers to their adoption exist for adolescents in child welfare institutions. Limited facility resources and a lack of caregiver support to fulfill adolescents’ physical and psychological needs can lead to low self-esteem and poor decision-making skills [6]. In addition, poor national health insurance coverage and inadequate government registration may lead to low awareness and limited access to health care services, further restricting HPBs [7].

This is especially true in the Indonesian context, which faces its own unique challenges. Because of family economic hardships, adolescents are placed in child welfare institutions with the expectation that they will receive better care and education than they did with their families [8]. However, the vast majority of institutions in Indonesia are private and lack adequate government oversight and support, meaning that they do not always comply with national care standards [9]. Consequently, adolescents living in such conditions become more susceptible to unhealthy behaviors, including smoking, poor sleep, low physical activity, and challenges in maintaining overall well-being [10]. Despite the recognized importance of HPBs during adolescence, research on the factors influencing HPBs among adolescents living in child welfare institutions in Indonesia remains limited.

The health promotion model (HPM) developed by Pender and Murdaugh [3] has frequently been used to examine the relationship between relevant factors and HPBs in the adolescent population. The HPM encompasses 3 interconnected domains: individual characteristics and experiences, behavior-specific cognitions and affect, and behavioral outcomes and HPBs. These serve as the end point, with the aim to achieve positive health outcomes. Individual characteristics such as previous behavior, health literacy, self-esteem, and sociodemographic factors significantly shape subsequent actions, with their impact varying depending on the specific behavior being considered. Behavior-specific cognitions and affect—including perceived benefits and barriers to action, perceived self-efficacy, activity-related affect, and interpersonal and situational influences—are highly motivational. These factors can be modified through targeted interventions, making their measurement essential for evaluating intervention effectiveness.

Previous studies have established an explicit connection between various factors that are considered determinants of HPBs among adolescents [11-14]. Perceived barriers to action were the strongest and most important predictor of HPBs, exhibiting a negative relationship—the lower the perceived barriers to action, the higher the HPBs among adolescents and young adults [11,12]. Perceived self-efficacy also plays a crucial role in determining HPBs, with higher perceived self-efficacy correlating with increased HPBs [13]. Higher perceived self-efficacy is also correlated with lower perceived barriers to action [14].

Social support also contributes to HPBs, with greater social support associated with higher levels of HPBs [15,16]. Moreover, social support can enhance a sense of belonging, which, in turn, increases perceived self-efficacy and further promotes HPBs among adolescents. This is especially critical during adolescence, when individuals begin to understand health-related information and make independent health decisions [17]. Health literacy not only reduces perceived barriers to action but also improves perceived self-efficacy [18,19]. Furthermore, individuals with higher health literacy are more likely to seek and receive greater social support from their networks [20].

Self-esteem as a key individual characteristic is a significant factor. Higher self-esteem reduces perceived barriers to action while simultaneously promoting greater perceived self-efficacy and social support [21-23].

### Objectives

This study aimed to identify the HPBs of adolescents in child welfare institutions in Indonesia and explore the determinants influencing these behaviors using the HPM. Given the relationships between the variables reviewed, this study examined health literacy and self-esteem as components of the individual characteristic domain and perceived barriers to action, perceived self-efficacy, and social support as elements of the behavior-specific cognitions domain. The HPBs examined as behavioral outcomes in this study were health responsibility, physical activity, nutrition, positive life perspective, interpersonal relationships, stress management, and spiritual health [24]. We specifically hypothesized that perceived barriers to action would be negatively correlated with HPBs, whereas perceived self-efficacy and social support would be positively correlated with HPBs. The findings of this study will provide insights into the challenges and opportunities for promoting HPBs in this population by identifying their determinants using structural equation modeling (SEM).

## Methods

### Study Design and Setting

This study used a cross-sectional design. Conducted in January 2024 and February 2024, this study took place in Malang, Indonesia. First, convenience sampling was used to select the child welfare institutions in Malang. A total of 17 child welfare institutions agreed to participate in this study. Inclusion criteria for this study were adolescents aged 13 to 18 years who had been living in child welfare institutions for at least 6 months and were willing to participate. Adolescents with developed mental or physical disabilities were excluded.

The sample size estimation for SEM is flexible. Several rules of thumb are commonly used, such as 10 cases per variable, 5 or 10 observations per estimated parameter, and a minimum sample size of 100 to 200 [25]. A sample size of 200 is often considered the gold standard in SEM and is widely used in studies [26,27]. Thus, this recommendation was applied while accounting for a 20% dropout rate, as reported in previous studies [28,29]. The required sample size for this study was calculated to be a minimum of 240.

Six research assistants collected data from participants. These assistants were third- and fifth-semester undergraduate nursing students who had completed the pediatric nursing theory and clinical practicum courses. The principal investigator provided instructions on the study’s purpose and managed the link to the questionnaire and other relevant procedures. During data collection, research assistants visited the institutions and presented the questionnaire to participants using tablets or notebooks, allowing them to complete the survey via the KoboToolbox platform. In addition, when participants needed help understanding any question, research assistants provided individualized explanations. Participants were given sufficient time to provide their answers, and completing the questionnaires took approximately 45 minutes.

Before the answers were submitted online, the research assistants verified that no responses were missing. Of the 280 adolescents who participated, 4 (1.4%) were excluded because they selected identical responses to every question. The final analysis included 276 participants.

### Ethical Considerations

The institutional review board of the Institute of Technology, Science, and Health RS Dr Soepraoen in Malang, Indonesia, approved this study (approval KEPK-EC-10/XII/2023). As adolescents are a vulnerable group requiring guardian consent for participation, the heads of the child welfare institutions provided written consent for the study, and all participants were asked to provide assent before data collection. The researchers ensured the privacy and confidentiality of participant data by not recording personal identifiers and by storing data in password-protected web-based storage accessible only to the research team. Participants who completed the questionnaire received a souvenir and a lunch box with an equivalent value of approximately US $1.50.

### General Participant Demographics

Demographic information about the participants included age (in years), sex (male or female), educational level (primary school, junior high school, senior high school, or undergraduate), orphan status (maternal orphan, paternal orphan, double orphan, or not orphan), and duration of residence in child welfare institutions (in years). In addition, the accreditation level of the child welfare institutions was recorded. Indonesian child welfare institutions receive accreditation ratings of A (excellent), B (good), or C (satisfactory) or are unaccredited, reflecting varying levels of compliance with national standards for child welfare services. Higher ratings indicate a greater capacity to provide quality care within an institution.

### HPB Measurement

In this study, HPBs refer to actions taken by adolescents to maintain and improve their health within the environment of a child welfare institution. The Adolescent Lifestyle Profile–Revised 2 [30] was used to measure HPBs. This instrument consists of 44 items across 7 domains: health responsibility, physical activity, nutrition, positive life perspective, interpersonal relationships, stress management, and spiritual health. The items were rated using a 4-point Likert scale (1=*never*, 2=*sometimes*, 3=*often*, and 4=*always*), with total possible scores ranging from 44 to 176. Higher scores indicate better HPBs among adolescents. The overall Cronbach α was 0.86 in this study.

### Health Literacy Measurement

Health literacy refers to adolescents’ ability to seek, understand, evaluate, and use health information to make informed decisions about their health. The Health Literacy Assessment Scale for Adolescents [31], consisting of 15 items, was used to measure health literacy. The instrument includes 3 subscales: communication, confusion, and functional health literacy. Items were rated on a 5-point Likert scale (1=*always*; 5=*never*), with total possible scores ranging from 15 to 75. Higher scores indicate greater health literacy. The Cronbach α for the scale was 0.70 in this study.

### Self-Esteem Measurement

Self-esteem refers to adolescents’ self-perception and evaluation of their own personal worth and abilities. The Rosenberg Self-Esteem Scale [32] adapted for the Indonesian context [33] was used to measure adolescents’ self-esteem. This instrument consists of 8 items rated on a 4-point Likert scale (1=*strongly disagree*, 2=*disagree*, 3=*agree*, and 4=*strongly agree*) for items 1, 2, 3, 6, and 8, with reverse scoring applied to items 4, 5, and 7. The scores range from 8 to 32, with higher scores indicating higher self-esteem. In this study, the Cronbach α was 0.68.

### Perceived Barrier to Action Measurement

Perceived barriers to action refer to adolescents’ perceptions of the barriers or obstacles that prevent them from engaging in HPBs. The Barriers to Health-Promoting Activities Scale [34], consisting of 18 items, was used to measure this perception. Items were rated on a 4-point Likert scale (1=*never*; 4=*routinely*), with total possible scores ranging from 18 to 72. Higher scores indicate greater perceived barriers to HPBs. In this study, the Cronbach α of the scale was 0.85.

### Perceived Self-Efficacy Measurement

Perceived self-efficacy refers to adolescents’ belief in their ability to perform and maintain HPBs. The Self-Rated Abilities for Health and Practices Scale–Adolescent Version [35] was used. This instrument includes 28 items across 4 subscales: nutrition, psychological well-being, exercise, and responsible health practices. This instrument was rated on a 5-point Likert scale (1=*cannot do at all*, 2=*a little*, 3=*somewhat*, 4=*mostly*, and 5=*certainly can do*), with total possible scores ranging from 28 to 140. Higher scores indicate greater perceived self-efficacy among adolescents. In this study, the Cronbach α was 0.92.

### Social Support Measurement

Social support refers to the emotional, instrumental, appraisal, and informational support that adolescents receive from caregivers, peers, and friends to help them engage in HPBs. The original Child and Adolescent Social Support Scale for Healthy Behaviors [36] is used to assess social support for healthy behaviors in children and adolescents from parents, teachers, classmates, close friends, and people in the school environment. For this study, this instrument was adapted to focus on 3 sources of support: caregivers, peers, and friends residing in child welfare institutions. This instrument consists of 36 items and 2 subscales: frequency and importance of social support. Frequency responses were rated on a 6-point Likert scale (1=*never*; 6=*always*), and importance responses were rated on a 3-point Likert scale (1=*not important*; 3=*very important*), with total possible scores ranging from 72 to 324. Higher scores indicate greater social support. The Cronbach α of the scale was 0.96 in this study.

### Translation Process

Among the instruments, the following 5 questionnaires—the Adolescent Lifestyle Profile–Revised 2, Health Literacy Assessment Scale for Adolescents, Barriers to Health-Promoting Activities Scale, Self-Rated Abilities for Health and Practices Scale–Adolescent Version, and Child and Adolescent Social Support Scale for Healthy Behaviors—were translated into Indonesian using forward and backward translation techniques before use [37]. To ensure the validity of the translated scales, a translation process was followed step by step. Two faculty members from the pediatric nursing department and one with a background in English literature who had worked in the health field for >5 years took part in the following translation process: (1) 2 translators independently translated the original instrument from English into Indonesian (version A); (2) a third translator, who was unfamiliar with the original instruments, translated version A back into English (version B); (3) all translators reviewed and compared version B with the original version to identify conceptual discrepancies, adapt culturally relevant terms, and develop a refined forward translation (version C); (4) for cognitive debriefing, 5 adolescents were interviewed to assess whether the translated items were easily understood and accurately conveyed the intended meaning of the original questionnaire (version D); and (5) the translators reconvened to review version D for conceptual equivalence and make final proofreading adjustments, resulting in the finalized version used in this study (version E).

### Statistical Analysis

SPSS Statistics (version 26.0; IBM Corp) was used to conduct descriptive analyses of variables, internal consistency tests, independent 2-tailed *t* tests, 1-way ANOVAs, Pearson correlations, and post hoc analyses. The SPSS Amos software (version 26.0; IBM Corp) was used to conduct path analyses examining the regression coefficients and the effects (direct, indirect, and total) among variables, as well as evaluating the fit of the structural model.

**Figure 1:**
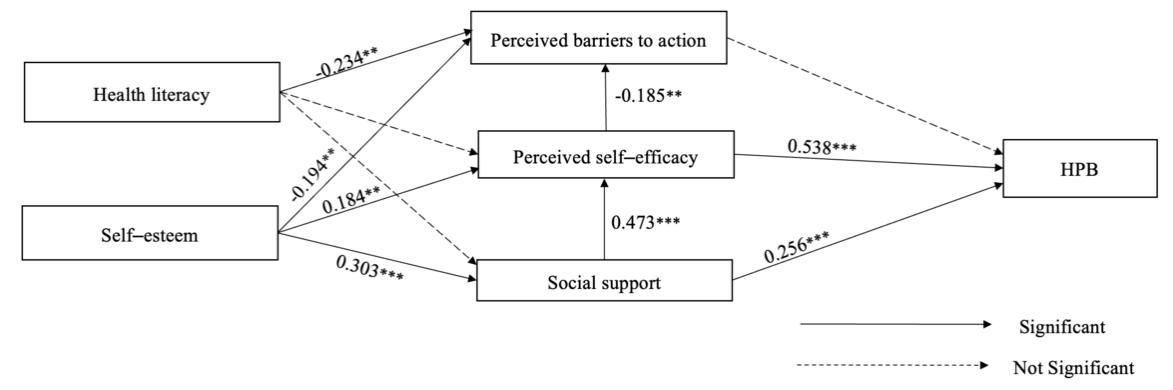
Path coefficient diagram of the final model of health-promoting behaviors (HPBs) among Indonesian adolescents living in child welfare institutions. *P<.05; **P<.01; ***P<.001.

The determinants of HPBs among adolescents living in child welfare institutions were identified using SEM. In this study, 6 variables included in the model were observed variables with a single indicator. The 2 exogenous variables—health literacy and self-esteem—served as independent variables (predictors) of the other observed variables in the model. The 4 endogenous variables included perceived barriers to action, perceived self-efficacy, social support, and HPBs. HPBs were identified as the outcome of the model, whereas perceived barriers to action, perceived self-efficacy, and social support were identified as mediators between the exogenous variables and HPBs. Figure 1 shows the hypothesized model for this study.

A structural equation model was also used to test the hypotheses. First, the model was identified, followed by an evaluation of its overall fit. The number of distinct sample moments in this model was 21, and the number of distinct parameters to be estimated was 18. Therefore, the *df* were calculated as 21−18=3. As the *df* were positive, a minimum was achieved, allowing for further testing [27]. Four tests were used to assess the goodness of fit: the Tucker-Lewis index (>0.90), comparative fit index (>0.90), standardized root mean square residual (<0.08), and root mean square error of approximation (<0.08) [38]. Bootstrapping analysis with 5000 samples and 95% bias-corrected CIs determined the significance of both indirect and direct effects.

## Results

### Participant Demographic and HPB Analysis

Of the 276 participants, 149 (54%) were female. Most (155/276, 56.2%) were aged 13 to 15 years, with a mean age of 15.3 (SD 1.7) years. In addition, 43.1% (119/276) were in senior high school, whereas 42.8% (118/276) were in junior high school. Approximately half (141/276, 51.1%) of the participants were not orphans, and 60.9% (168/276) had been living in child welfare institutions for 1 to 3 years, with a mean duration of 3.4 (SD 2.5) years. Table 1 presents participants characteristics and comparison of HPBs.

**Table 1:** Participant characteristics and comparison of health-promoting behaviors (HPBs) between groups (N=276).

Variable and category			Participants, n (%)		Variable mean (SD)		HPBs—ALP-R2^a^ (score range 44-176), mean (SD)		*P* value
**Age (y)**					15.3 (1.7)				.03
	13-15	155 (56.2)				122.0 (15.0)			
	16-18	121 (43.8)				125.0 (12.6)			
**Sex**					—^b^				.40
	Male	127 (46)				124.2 (14.3)			
	Female	149 (54)				122.5 (13.9)			
**Educational level (school)**					—				.04
	Primary	28 (10.1)				116.8 (13.1)			
	Junior high school	118 (42.8)				123.6 (14.7)			
	Senior high school	119 (43.1)				125.0 (13.4)			
	College	11 (4)				118.3 (12.5)			
**Orphan status**					—				.73
	Not orphan	141 (51.1)				124.2 (14.1)			
	Maternal	23 (8.3)				120.9 (13.9)			
	Paternal	88 (31.9)				123.8 (14.5)			
	Double	24 (8.7)				119.0 (11.5)			
**Duration of residence in child welfare institutions (y)**					3.4 (2.5)				.03
	1-3	168 (60.9)				123.6 (14.0)			
	4-5	77 (27.9)				125.2 (14.1)			
	>5	31 (11.2)				117.0 (13.3)			
**Accreditation level of institutions**					—				.02
	A	20 (7.2)				117.3 (15.0)			
	B	125 (45.3)				125.5 (13.4)			
	C	51 (18.5)				118.2 (13.9)			
	Unaccredited	80 (29)				124.7 (14.0)			

^a^ALP-R2: Adolescent Lifestyle Profile–Revised 2.

^b^Not applicable.

HPB scores were significantly different by age group (*P*=.03), educational level (*P*=.04), duration of residence in child welfare institutions (*P*=.03), and accreditation level of institutions (*P*=.02). On the basis of the post hoc analysis, adolescents aged 13 to 15 years had lower HPB scores than adolescents aged 16 to 18 years (*P*=.03). Primary school students had lower HPB scores than college students (*P*=.03). HPB scores were higher among those living in child welfare institutions for 1 to 3 years and 4 to 5 years than among those living in them for >5 years (*P*<.05). Adolescents in level B institutions had higher HPB scores than those in level C institutions (*P*=.02), and adolescents living in unaccredited institutions had higher HPB scores than those living in level C institutions (*P*=.03).

### Correlation Between Major Variables

The correlation analysis showed that HPBs had a significant positive correlation with perceived self-efficacy (*r*=0.66; *P*<.001), social support (*r*=0.53; *P*<.001), health literacy (*r*=0.13; *P*=.03), and self-esteem (*r*=0.31; *P*<.001). Conversely, HPBs had a significant negative correlation with perceived barriers to action (*r*=−0.15; *P*=.01). Table 2 presents the results of the correlation analysis between variables.

**Table 2:** Correlation analysis between variables.

	Health literacy	Self-esteem	Perceived barriers to action	Perceived self-efficacy	Social support
**Health literacy**					
r	1	0.26	−0.31	0.16	0.02
*P* value	—^a^	<.001	<.001	<.001	.76
**Self-esteem**					
r	0.26	1	−0.32	0.35	0.29
*P* value		—	<.001	<.001	<.001
**Perceived barriers to action**					
r	−0.31	−0.32	1	−0.29	−0.23
*P* value	<.001	<.001	—	<.001	<.001
**Perceived self-efficacy**					
r	0.16	0.35	−0.29	1	0.53
*P* value	.01	<.001	<.001	—	<.001
**Social support**					
r	0.02	0.29	−0.23	0.53	1
*P* value	.76	<.001	<.001	<.001	—
**HPBs^b^**					
r	0.13	0.31	−0.15	0.66	0.53
*P* value	.03	<.001	.01	<.001	<.001

^a^Not applicable

^b^HPB: health-promoting behavior.

### HPBs and Significant Determinants

The model in this study indicated a good fit (Tucker-Lewis index=0.947; comparative fit index=0.989; standardized root mean square residual=0.000; root mean square error of approximation=0.070). The results of the SEM analysis are presented in Table 3. In the HPB pathway, only perceived self-efficacy (β=0.538; *P*<.001) and social support (β=0.256; *P*<.001) had significant positive correlations with HPBs. These 2 variables explained 47.9% of the variance in HPBs. In the perceived barriers to action pathway, perceived self-efficacy (β=−0.185; *P*<.01), health literacy (β=−0.234; *P*<.001), and self-esteem (β=−0.194; *P*<.01) showed significant negative correlations with perceived barriers to action, collectively explaining 18.8% of its variance. In the perceived self-efficacy pathway, only self-esteem (β=0.184; *P*<.001) and social support (β=0.473; *P*<.001) had significant positive correlations, explaining 32.8% of its variance. In the social support pathway, only self-esteem (β=0.303; *P*<.001) had a significant positive correlation with social support, explaining 8.6% of its variance.

**Table 3:** Effect coefficients of the health-promoting behavior (HPB) model among adolescents living in child welfare institutions.

Dependent and independent variable			Direct effect (β)		Indirect effect (β)		Total effect (β)		SMC^a^	
**HPBs**										0.479
	Perceived barriers to action	0.065		—^b^		0.065				
	Perceived self-efficacy	0.538^c^		−0.012		0.526^c^				
	Social support	0.256^c^		0.249^c^		0.505^c^				
**Perceived barriers to action**										0.188
	Perceived self-efficacy	−0.185^d^		—		−0.185^d^				
	Social support	—		−0.088^d^		−0.088^d^				
	Health literacy	−0.234^d^		−0.013		−0.247^c^				
	Self-esteem	−0.194^d^		−0.061^d^		−0.254^c^				
**Perceived self-efficacy**										0.328
	Health literacy	0.098		−0.029		0.069				
	Self-esteem	0.184^d^		0.143^c^		0.327^d^				
	Social support	0.473^c^		—		0.473^c^				
**Social support**										0.086
	Health literacy	−0.061		—		−0.061				
	Self-esteem	0.303		—		0.303^c^				

^a^SMC: squared multiple correlation.

^b^Not applicable.

^c^*P*<.001.

^d^*P*<.01.

In addition, there was a significant indirect effect of self-esteem on HPBs through perceived self-efficacy (β=0.099; *P*<.001), social support (β=0.078; *P*<.001), and the combination of social support and perceived self-efficacy (β=0.077; *P*<.001). The final model of HPBs among adolescents living in child welfare institutions is shown in Figure 1.

## Discussion

### Principal Findings

This study explored the determinants of HPBs among adolescents living in Indonesian child welfare institutions. It primarily found significant differences in adolescents’ HPBs based on age group, educational level, duration of stay in child welfare institutions, and the accreditation level of the institutions. Older adolescents and those with higher educational levels demonstrated better HPBs, which may be attributed to greater responsibility and autonomy [39]. Interestingly, adolescents who had lived in child welfare institutions for >5 years had significantly lower HPBs than those who had lived there for shorter periods, such as 1 to 3 years and 4 to 5 years. This may be due to adaptation to regular daily schedules, although prolonged stays without proper knowledge might lead to the normalization of poor HPBs. In addition, adolescents who have just entered these institutions might receive more attention and support from caregivers and peers than long-term residents. These findings provide valuable insights into the necessity and timing of delivering internal education to promote healthy behaviors. In terms of accreditation levels, adolescents living in level B institutions showed significantly higher HPBs than those in level C institutions. However, adolescents living in level A institutions did not demonstrate better HPBs than those living in level B or C institutions. Higher-level institutions are assumed to have better access to services and more structured systems than lower-level ones, potentially offering adolescents more resources to engage in good HPBs. These unexpected findings might be due to the small sample size of level A institutions and the fact that the accreditation process primarily focuses on environmental and human support factors rather than the promotion of health behaviors among the residents. This aspect is suggested as a criterion in the future accreditation process for Indonesian child welfare institutions.

Through the SEM analysis, this study found that perceived self-efficacy and social support together accounted for 47.9% of adolescents’ HPBs. HPBs are key to happiness and a good quality of life, and self-efficacy increases HPBs at every stage of life [40]. When individuals have confidence in their ability to perform HPBs, they are more likely to engage in these behaviors [4]. Among adolescents in child welfare institutions, social support appears to play a crucial role in the development of good HPBs, helping bring more structure to their daily activities.

This study challenges existing frameworks by highlighting that perceived barriers to action were not significant to HPBs in this context, providing insights into the unique needs of these adolescents. This finding aligns with findings from Thailand [41]. This could be attributed to the unique environment of child welfare institutions, where structured activities and support systems mitigate the barriers typically faced by adolescents living with their families. The organized nature of these institutions—with regulated schedules and caregiver supervision—can encourage adolescents to engage in HPBs, often leaving health decision-making to caregivers and the institution. This is supported by the fact that most participants in this study (196/276, 71%) lived in accredited child welfare institutions (level A, B, or C), which have already met the minimum national care standards for children.

Interestingly, health literacy did not correlate with perceived self-efficacy in this study. This finding differs from those of studies conducted in Australia, China, Turkey, and Germany, which found a positive correlation between health literacy and adolescent self-efficacy [18]. This discrepancy may be due to adolescents in child welfare institutions having moderate health literacy but lacking confidence in performing HPBs. In general, adolescents are not fully independent at this stage and often lack autonomy in their activities, as well as practical experience in carrying them out [42].

In addition, this study identified the link between self-esteem, social support, and perceived self-efficacy and HPBs, a connection that has emerged in recent literature on this unique population. It may provide a foundation for health professionals to develop interventions that address HPBs by improving self-esteem and enhancing social support for targeted adolescents such as those in this study.

### Comparison With Prior Work

No previous studies—to our knowledge—have examined HPBs among adolescents living in child welfare institutions. Much of the existing research has focused on healthy adolescent populations, with studies conducted in Turkey [43], Portugal [44], Iran [45], and India [46]. However, this study explored HPBs among a specific group of adolescents—those living in child welfare institutions—which has been understudied in Indonesia and other countries. This study also highlights several factors under the HPM that influence HPBs, providing a comprehensive understanding of the determinants of these behaviors.

This study found that self-esteem had a significant indirect effect on HPBs through perceived self-efficacy and social support. This supports previous studies indicating that higher self-esteem improves perceived self-efficacy, which is crucial for problem-solving and adopting HPBs [47,48]. Individuals with high self-esteem tend to have greater perceived self-efficacy in problem-solving [49,50].

This study also found that only self-esteem showed a significant positive correlation with social support and self-efficacy and indirectly affected HPBs. Higher self-esteem leads to more active social engagement, thereby increasing perceived social support [51]. Interestingly, self-esteem explained only 8.6% of the variance in social support. Social support is a complex and multidimensional construct [52], and we assessed it only from caregivers, peers, and friends living in child welfare institutions. In addition, only 8.7% (24/276) of the participants were double orphans, indicating that most participants still had family members with whom they maintained contact, which may also influence their social support. The limitation of the social support measurement may have affected the strength of the observed relationship, and future studies should investigate the role of family support among adolescents living in child welfare institutions who are not double orphans.

Furthermore, self-esteem was found to be moderate in this study, which is consistent with previous research indicating that adolescents living in child welfare institutions generally exhibit low to moderate levels of self-esteem [53]. Hence, interventions to improve self-esteem could enhance social support and, consequently, HPBs. In child welfare institutions, fostering a nurturing environment that enhances self-esteem and interpersonal relationships can be used to boost adolescents’ perceived self-efficacy, thereby promoting positive HPBs.

This study also confirmed that social support is positively correlated with perceived self-efficacy, which helps reduce perceived barriers to action. These findings align with those of a study from the United States that showed that higher perceived self-efficacy reduces perceived barriers to physical activity—a component of HPBs [54]. Greater social support leads to higher perceived self-efficacy and fewer perceived barriers. Given the limited studies on this topic—especially among adolescents—this study provides valuable insights into the relationships among self-esteem, social support, perceived self-efficacy, and perceived barriers to action.

The key finding of this study was the nuanced relationship between health literacy and HPBs among adolescents in child welfare institutions. Surprisingly, health literacy did not significantly influence perceived self-efficacy or social support, suggesting that the structured environment of these institutions provided consistent information and support, reducing reliance on individual health literacy. In addition, adolescents are still in the process of developing their health-related knowledge, and the lack of health education interventions within child welfare institutions may limit the impact of health literacy on their ability to benefit from social support. However, lower health literacy significantly increased perceived barriers to action, indicating that, even within a supportive environment, understanding health information is crucial for overcoming challenges. The structured environment of the institutions mitigated the negative impact of these perceived barriers to HPBs, suggesting that consistent support and resources can buffer the effects of perceived obstacles. These findings underscore the need for interventions that enhance health literacy, empowering adolescents to overcome barriers while leveraging the supportive nature of structured environments in child welfare institutions to promote healthier behaviors and improve adolescents’ overall well-being.

### Clinical and Policy Implications

The findings of this study highlight the need for policies specific to the improvement of HPBs in adolescents living in child welfare institutions in Indonesia. The large number of unrecorded child welfare institutions hinders the provision of nursing interventions to these children and adolescents. Coordination between the Ministry of Social Affairs and Ministry of Health is essential, starting with data collection from child welfare institutions and regular monitoring of the health and welfare of children in these institutions. This will enable health professionals to provide optimal health services and interventions for these adolescents.

While school-based interventions are often considered effective, a review found that these interventions were insufficient for improving HPBs [55]. This is consistent with conditions in Indonesia, where the concept of school nurses or other health professionals at school is not widespread. Only a few private schools have health care professionals who work full time or part time. This may lead to inadequate health interventions in schools. Public health centers are the closest access points for adolescents. Public health centers conduct annual health screenings for students and have a youth health care program that usually provides health services and education for schools at least twice a year. Within these programs, health professionals can also administer HPB assessments and provide education for adolescents in schools.

It has also been suggested to extend interventions beyond the school setting into the home [55]. This is feasible in Indonesia. This study found that social support from caregivers and friends in child welfare institutions was more significant than that from peers. Unfortunately, public health centers do not have specific programs for child welfare institutions. However, they have an Islamic boarding school health care center (*poskestren*) program, which focuses on health services to Islamic boarding schools. Public health centers can include child welfare institutions in this program, conducting health interventions for adolescents, caregivers, and other children in these institutions, thus improving HPBs among adolescents in Indonesia.

### Study Limitations

Despite our best efforts, a few limitations exist in this study. First, convenience sampling methods were used in a single city, so generalizing the findings should be done with caution. Second, this study had a cross-sectional design, which requires careful interpretation of causality as bidirectional relationships between variables may exist. Therefore, we encourage future studies to use a longitudinal design to gain a deeper understanding of HPBs among adolescents living in child welfare institutions over time. Third, because half of the participants had at least one parent, social support from parents—which was not assessed—may have influenced the level of social support they received. Therefore, we suggest that future studies assess social support from parents and its potential impact on HPBs. Fourth, exploratory factor analysis was conducted; however, no confirmatory factor analyses were conducted on the translated instruments, which may have impacted the validity of the measures. Finally, although the research assistants were trained to ensure consistent interpretation of the instruments, periodic meetings were not held to calibrate interpretations or address issues that arose during data collection. This may have affected the reliability and accuracy of the data collection process.

### Conclusions

On the basis of the HPM and testing using SEM analysis, both perceived self-efficacy and social support directly affected HPBs, whereas self-esteem indirectly affected HPBs among adolescents in child welfare institutions in Indonesia. In addition, health literacy only influenced perceived barriers to action, but these perceived barriers to action did not contribute to HPBs. The key findings of our study contribute to the development of health interventions aimed at improving self-efficacy and social support by enhancing self-esteem, ultimately leading to increased HPBs among adolescents living in child welfare institutions in Indonesia. We hope that these interventions will lead to improved health outcomes and quality of life for these individuals as they transition into adulthood and move from child welfare institutions to the broader community.

## Abbreviations

HPB: health-promoting behavior
HPM: health promotion model
SEM: structural equation modeling

## Data Availability

The datasets generated or analyzed during this study are available from the corresponding author on reasonable request.
